# Surgical resection of a rapidly growing pulmonary spindle cell carcinoma by robot-assisted thoracoscopic surgery: a case report

**DOI:** 10.1186/s40792-021-01305-5

**Published:** 2021-10-10

**Authors:** Akihiro Koen, Hideyuki Maeda, Yoji Nagashima, Masato Kanzaki

**Affiliations:** 1 Department of Thoracic Surgery, Tokyo Women’s Medical University, 8-1 Kawada-cho, Shinjuku-ku, Tokyo, 162-8666 Japan; 2 Department of Surgical Pathology, Tokyo Women’s Medical University, Tokyo, Japan

**Keywords:** Lung cancer, Spindle cell carcinoma, Robot-assisted thoracoscopic surgery, Lymph node dissection

## Abstract

**Background:**

Pulmonary spindle cell carcinoma (PSCC) is an extremely rare tumor that is highly malignant and fast-growing. As chemotherapy and radiation therapy are ineffective, early surgical resection is effective for PSCC.

**Case presentation:**

A 70-year-old woman with rheumatoid arthritis was referred to our hospital with an abnormal shadow. Chest computed tomography revealed a 33-mm-wide lobular mass in the right upper lobe. She was diagnosed with non-small cell lung cancer by bronchoscopic smear cytology. Although staging evaluation indicated stage IIIB (T3N2M0) disease, she required continued administration of immunosuppressants and prednisolone for rheumatoid arthritis. Therefore, robot-assisted thoracoscopic surgery (RATS) right upper lobectomy followed by lymph node dissection was performed without preoperative chemotherapy and radiotherapy. Pathological findings revealed PSCC.

**Conclusions:**

We report a very rare case of pulmonary spindle cell carcinoma, successfully resected with RATS.

## Background

Pulmonary spindle cell carcinoma (PSCC) is a sarcoma-like carcinoma consisting of only spindle-shaped tumor cells according to the 2015 World Health Organization (WHO) histological classification of lung cancer [1]. Because of poor response to chemotherapy and radiotherapy and poor patient prognosis, early surgical resection is effective for PSCC. We report a case of rapidly growing PSCC that was resected by robot-assisted thoracoscopic surgery (RATS).

## Case presentation

**Fig. 1:**
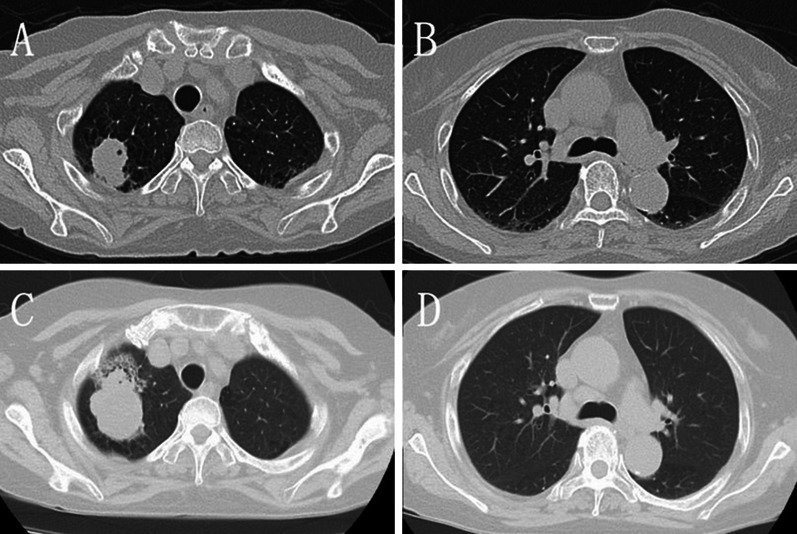
**A** Chest computed tomography (CT) scan shows a lobular mass of 33 mm located in the right upper lobe and **B** lymph node enlargement in station #4R. **C** Chest CT scan, taken 1 month after consultation, shows that the tumor diameter increased to 52 mm and **D** lymph node enlargement in station #4R

**Fig. 2:**
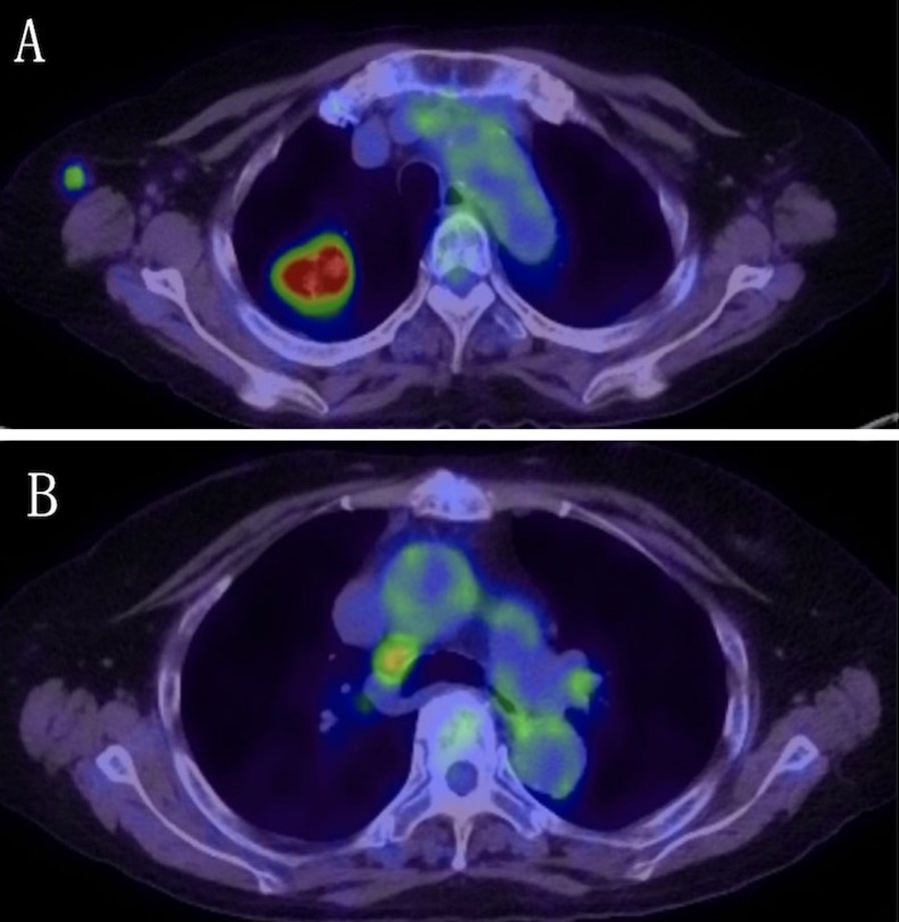
**A** 18F-fluorodeoxyglucose (FDG) positron emission tomography scan shows FDG accumulation with a maximum standardized uptake value (SUVmax) of 11.78 in the mass and **B** FDG accumulation with a SUVmax of 3.48 in the right lower paratracheal lymph node (station #4R)

**Fig. 3:**
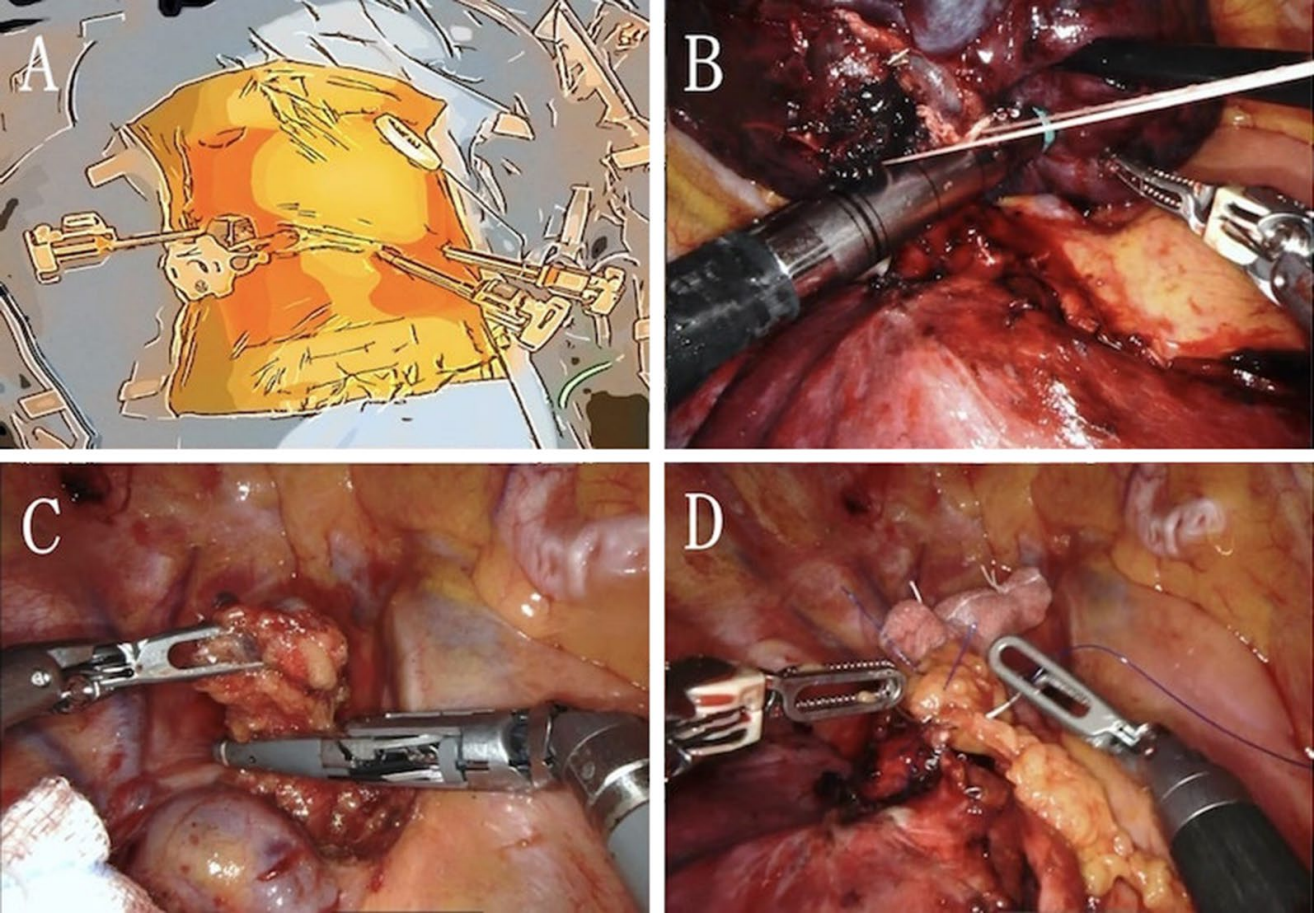
**A** Four-port incisions and a CO_2_ insufflation combined assistant port before docking the robot. The patient’s head is at the top of the picture. **B** Robot-assisted thoracoscopic right upper lobectomy followed by lymph node dissection is performed. **C** Upper mediastinum (station #4R) dissection. **D** Bronchial stump covered by a pericardial fat pad

**Fig. 4:**
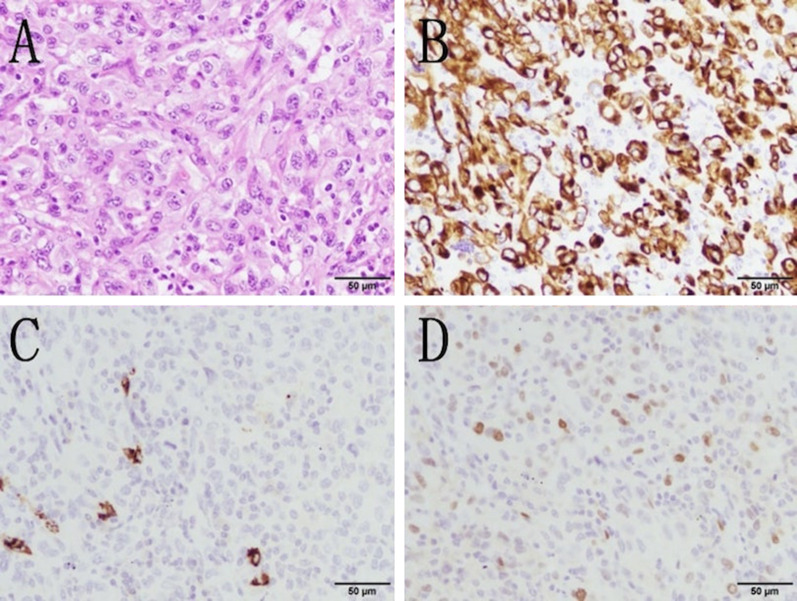
Histopathological findings. **A** Hematoxylin and eosin staining shows densely growing spindle-shaped cells with weak connectivity in some areas. The binding weak fusiform cells multiplied thickly and showed no differentiation between adenocarcinoma and squamous cell carcinoma. Immunohistologically, the tumor cells stained positive for **B** cytokeratin (AE1/AE3) and negative for **C** napsin A and **D** TTF-1

A 70-year-old woman with rheumatoid arthritis was referred to our hospital with an abnormal shadow in the right upper lung field detected by chest roentgenogram. Laboratory tests showed that only the level of pro-gastrin-releasing peptides was elevated (147.8 pg/mL). In pulmonary function tests, obstructive ventilatory disorders and low diffusing capacity were revealed. Chest computed tomography (CT) revealed a lobular mass of 33 mm in maximum diameter at the apex of the right upper lobe (Fig. 1A). 18F-fluorodeoxyglucose (FDG) positron emission tomography revealed FDG accumulation with maximum standardized uptake values of 11.78 in the mass and 3.48 in the right lower paratracheal lymph node (#4R) (Fig. 2A, B). Magnetic resonance imaging of the head did not reveal any findings suggestive of brain metastases. She was diagnosed with non-small cell lung cancer by bronchoscopic smear cytology. One month later, chest CT revealed that the tumor diameter had increased to 52 mm (Fig. 1B). Although staging evaluation indicated stage IIIB (T3N2M0) disease, due to rheumatoid arthritis, she required continued administration of immunosuppressants and prednisolone. RATS right upper lobectomy followed by lymph node dissection was performed, and the bronchial stump was covered with a pericardial fat pad for long-term immune support and prednisolone (Fig. 3A, B). Histopathologically, the tumor had a maximum diameter of 51 mm and a cavitary necrotic lesion. Hematoxylin and eosin staining revealed densely growing spindle-shaped cells (Fig. 4A). Immunohistochemically, except for cytokeratin (AE1/AE3) (Fig. 4B), negative staining for leukocyte-common antigen, α-smooth muscle actin, S-100 protein, thyroid transcription factor-1 (Fig. 4C), and napsin A were shown. Based on these findings, the final diagnosis was PSCC. There was no lymph node metastasis or pleural infiltration, and the pathological stage was stage IIB (T3N0M0). The postoperative course was uneventful.

## Discussion

According to the 2015 WHO histological classification of lung tumors, pulmonary sarcomatoid carcinoma includes pleomorphic carcinoma, PSCC, giant cell carcinoma, carcinosarcoma, and pulmonary blastoma [1]. Sarcomatoid carcinomas are mostly polymorphic carcinomas, accounting for 0.3 to 1.3% of all lung tumors, especially PSCC [1–7].

After the widespread use of the 2015 WHO histological classification of lung tumors, reports on PSCC have increased. Many reports confirm that cytology alone or endoscopic/percutaneous biopsy is very difficult to prove, as only spindle-shaped tumor cells need to be confirmed for diagnosis, and a definitive diagnosis is rarely obtained before surgery [1–7].

Pathological findings are that the spindle-shaped cells show a sarcomatoid, bundle-like, and flower-like arrangement and are composed only of spindle-shaped tumor cells, and immunostaining is useful for diagnosis [8]. Nakajima et al. reported that both epithelial markers CAM5.2 and AE1/AE3 were positive or one of them was positive in all 37 cases of sarcomatoid carcinoma [9]. It has been shown that the positive rate of epithelial markers was high in PSCC, while the positive rates of TTF-1 and napsin A which is a marker for lung adenocarcinoma were low [6]. In this case, both CAM5.2 and AE1/AE3 had positive staining, while TTF-1 and napsin A had negative staining and the tumor component only had spindle-shaped tumor cells.

The average age of onset of PSCC is 60 years; it is more common in men than in women and is strongly associated with smoking, and recurrence is likely to occur early after surgery [10]. The 5-year survival rate for all sarcomatoid lung cancers is as low as about 20% [1, 2], because rapid tumor progression leads to early recurrence and metastasis after surgery. As several reports have concluded that the effects of chemotherapy and radiation therapy are inadequate in inoperable cases, early diagnosis and treatment by surgical resection are important in PSCC [2–5, 7, 9, 10]. Due to rheumatoid arthritis, the patient with clinical single N2 lymph node metastasis required continued administration of immunosuppressants and prednisolone. Therefore, RATS right lobectomy was performed without preoperative chemotherapy and radiotherapy. Multiple studies show the effectiveness of RATS for lymph node dissection [11–13]. RATS has been reported to have a higher accuracy of lymph node dissection and less local recurrence of lymph nodes than thoracotomy or video-assisted thoracoscopic surgery [11, 13]. Fortunately, she has no lymph node metastasis, and the pathological stage was down and stage IIB. Postoperative follow-up is underway.

## Conclusions

We report a very rare case of PSCC, successfully resected with RATS. RATS enables accurate manipulation in thoracic cavity and may allow to perform more safe and less invasive thoracic surgery.

## Data Availability

Not applicable.

## Abbreviations

CT: Computed tomography
PSCC: Pulmonary spindle cell carcinoma
FDG: 18F-fluorodeoxyglucose
WHO: World Health Organization
RATS: Robot-assisted thoracoscopic surgery
TTF-1: Thyroid transcription factor-1
